# PPARα L162V underlies variation in serum triglycerides and subcutaneous fat volume in young males

**DOI:** 10.1186/1471-2350-8-55

**Published:** 2007-08-16

**Authors:** Julieta Uthurralt, Heather Gordish-Dressman, Meg Bradbury, Carolina Tesi-Rocha, Joseph Devaney, Brennan Harmon, Erica K Reeves, Cinzia Brandoli, Barbara C Hansen, Richard L Seip, Paul D Thompson, Thomas B Price, Theodore J Angelopoulos, Priscilla M Clarkson, Niall M Moyna, Linda S Pescatello, Paul S Visich, Robert F Zoeller, Paul M Gordon, Eric P Hoffman

**Affiliations:** 1 Research Center for Genetic Medicine, Children's National Medical Center, Washington DC, 20010, USA; 2 Obesity, Diabetes and Aging Research Center, College of Medicine, University of South Florida, 12901 Bruce B. Downs Blvd, Tampa, Florida 33612, USA; 3 Division of Cardiology, Henry Low Heart Center, Hartford Hospital, Hartford, CT, 06102, USA; 4 Department of Diagnostic Radiology, Yale University School of Medicine, New Haven, CT 06520, USA; 5 Center for Lifestyle Medicine and Department of Health Professions, University of Central Florida, Orlando FL 32816, USA; 6 Department of Exercise Science, Totman Building, University of Massachusetts, Amherst, MA, 01003, USA; 7 Department of Sport Science and Health, Dublin City University, Dublin 9, Ireland; 8 School of Allied Health, University of Connecticut, Storrs, CT 06269, USA; 9 Human Performance Laboratory, Central Michigan University, Mount Pleasant, Mich. 48859, USA; 10 Department of Exercise Science and Health Promotion, Florida Atlantic University, Davie, FL 33314, USA; 11 Division of Exercise Physiology, School of Medicine, West Virginia University, Morgantown WV 26506, USA

## Abstract

**Background:**

Of the five sub-phenotypes defining metabolic syndrome, all are known to have strong genetic components (typically 50–80% of population variation). Studies defining genetic predispositions have typically focused on older populations with metabolic syndrome and/or type 2 diabetes. We hypothesized that the study of younger populations would mitigate many confounding variables, and allow us to better define genetic predisposition loci for metabolic syndrome.

**Methods:**

We studied 610 young adult volunteers (average age 24 yrs) for metabolic syndrome markers, and volumetric MRI of upper arm muscle, bone, and fat pre- and post-unilateral resistance training.

**Results:**

We found the PPARα L162V polymorphism to be a strong determinant of serum triglyceride levels in young White males, where carriers of the V allele showed 78% increase in triglycerides relative to L homozygotes (LL = 116 ± 11 mg/dL, LV = 208 ± 30 mg/dL; p = 0.004). Men with the V allele showed lower HDL (LL = 42 ± 1 mg/dL, LV = 34 ± 2 mg/dL; p = 0.001), but women did not. Subcutaneous fat volume was higher in males carrying the V allele, however, exercise training increased fat volume of the untrained arm in V carriers, while LL genotypes significantly decreased in fat volume (LL = -1,707 ± 21 mm^3^, LV = 17,617 ± 58 mm^3 ^; p = 0.002), indicating a systemic effect of the V allele on adiposity after unilateral training. Our study suggests that the primary effect of PPARα L162V is on serum triglycerides, with downstream effects on adiposity and response to training.

**Conclusion:**

Our results on association of PPARα and triglycerides in males showed a much larger effect of the V allele than previously reported in older and less healthy populations. Specifically, we showed the V allele to increase triglycerides by 78% (p = 0.004), and this single polymorphism accounted for 3.8% of all variation in serum triglycerides in males (p = 0.0037).

## Background

Metabolic syndrome is considered to be a pre-diabetic state, with abnormal values for triglycerides, HDL, adiposity, blood pressure, and insulin resistance [1]. Twin and family studies have demonstrated the prominent influence of genetic factors on metabolic syndrome sub-phenotypes [2,3]. To date, most research aimed to identify these genetic underpinnings have been done in elderly or diseased populations. Less progress has been made on the identification of initial risk factors such as lipid levels and insulin resistance in young individuals. With regards to these sub-phenotypes, it is known that the genetic factors in an aged population may be different than those in a younger population. For example, a longitudinal twin study has reported that only 40% of the genetic factors affecting BMI are shared at the age of 20 y and 40 y [4].

We have previously described a genetic association study of university-age young adults, with measurement of baseline strength and tissue volumes of the upper arm, then response to a 12 wk unilateral resistance training of the non-dominant arm [5-8]. We chose the non-dominant arm to minimize environmental influences (use), and thus maximize sensitivity for detecting genetic influences. We recently expanded the study to include baseline metabolic syndrome measures. In this study, we used upper arm subcutaneous fat as a surrogate marker for adiposity, although visceral fat is more highly studied as a measure of obesity. Here, our goal was to test the effect of the PPARα gene polymorphism (L162V) on a young adult volunteer population, and to begin to better dissect the effects of PPARα genotype on the earliest phenotypes.

Peroxisome-proliferator-receptor-alpha (PPARα) is involved in adipocyte differentiation, lipid and lipoprotein metabolism. Studies in mice have shown that PPARα-deficient animals were unable to metabolize lipids and develop late onset obesity even when kept on a stable diet [9,10]. PPARα is activated by circulating fatty acids, and peroxisome proliferator receptor agonists such as hypolipidemic drugs (fibrates), commercially used plasticizers, synthetic fatty acids, steroid hormones, herbicides and pesticides [11]. Activation of the receptor results in the increased expression of genes involved in lipid oxidation, lipoprotein metabolism, inhibition of vascular inflammation and adipocyte differentiation through binding to peroxisome proliferator response elements (PPRE) in the DNA sequence of target genes [12]. In summary the activation of PPARα stimulates fatty acid transportation and oxidation primarily in liver and muscle reducing the storage of fat in adipocytes. Thus, PPARα is a strong candidate gene for the genetic component of metabolic syndrome.

The PPARα gene has been screened for common polymorphic variations. The most studied variant is a missense mutation in exon 5 that results in a nonconservative amino acid substitution, leucine 162 valine (L162V). The V allele is Leucine 162 is located within the DNA binding domain and is highly conserved among species [13,14]. Functional studies of the PPARα L162V polymorphism have shown differences in the activity of the receptor depending on the ligand concentration. At low ligand concentrations the activity of the receptor was lower for the V isoform than the L isoform, whereas at high ligand concentrations the opposite occurred (V activity decreased relative to L) [15]. The L162V polymorphism has been previously associated with dyslipidemia [16-21], and some measures of adiposity [22,23]. However, some of these studies showed opposite findings or negative associations. Many factors may contribute to an inability to replicate genetic association studies. First, differences in the population studied with most published studies focusing on older or unhealthy populations. Second, different methods of measuring both regional and total adiposity are used, and each of these methods has well-documented issues concerning reliability of measure, sensitivity, and specificity for measuring fat.

The aim of this study was to identify the role that PPARα L162V polymorphism may play in the variability of serum lipid concentration and variations in subcutaneous fat volume and BMI in 610 young healthy volunteers (average age 24 years). Fat volume was determined using semi-automated three-dimensional reconstruction analysis software. We tested the PPARα L162V polymorphism against our volumetric MRI measures and variations in serum lipid profiles. We found strong statistical support for sex-specific effects of PPARα genotype with plasma triglyceride, HDL, and BMI. The effect of PPARα genotype was much stronger in our young adult population than previously reported for older populations, suggesting that younger populations may afford greater sensitivity in identification of the genetic underpinnings of metabolic syndrome sub-phenotypes.

## Methods

### Study overview and subjects

The study population was derived from a multicenter, NIH funded study designed to identify genetic factors that dictate baseline bone, muscle and fat volume and the variability in response to exercise training. The study design protocol has been described in detail elsewhere [24], and preliminary reports of genetic associations with muscle strength and size have been reported [5-8]. Briefly, 1,300 men and women, average age 24 (range 18 – 40 yrs) were recruited by one of the 8 centers (University of Massachusetts Amherst, University of Connecticut, Dublin University (Ireland), Florida Atlantic University, Hartford Hospital, University of Central Florida, West Virginia University, Central Michigan University). Participants were excluded if they: were <18 y or >40 y; used medications known to affect skeletal muscle such as corticosteroids; had any restriction of activity; had chronic medical conditions such as diabetes; had metal implants in arms, eyes, head, brain, neck, or heart which would prohibit MRI testing; had performed strength training or employment requiring repetitive use of the arms within the prior 12 months; consumed on average >2 alcoholic drinks daily; or had used dietary supplements reported to build muscle size/strength or to cause weight gain such as protein supplements, creatine, or androgenic precursors. Subjects were asked to maintain their normal dietary intake for the duration of the study. Written informed consent was obtained from each volunteer. The study was approved by the Children's National medical Center Institutional Review Board (protocol #2449), and was in compliance with the Helsinki Declaration.

We limited this current study to 610 Caucasians that had values for entry metabolic syndrome markers, completed pre- and post-MRI measurements, or both (table 1).

**Table 1:** Demographic characteristics of Caucasian FAMuSS cohort

**Characteristic**	**Females**		**Males**	
	
	**N**	**Mean ± SD**	**N**	**Mean ± SD**
Age (years)	378	24.12 ± 7.75	223	26.48 ± 11.70*
Baseline body mass (lbs.)	378	144.35 ± 28.17	223	180.19 ± 37.39 **
Baseline height (in.)	378	64.96 ± 2.63	223	69.94 ± 2.76 **
Baseline BMI	378	24.06 ± 4.66	223	25.88 ± 5.12 **
Post-exercise body mass (lbs.)	378	145.40 ± 28.03	223	181.04 ± 37.15 **
Post-exercise BMI	378	24.22 ± 4.62	223	25.98 ± 5.06 **
Fasting glucose (mg/dL)	378	85.59 ± 7.60	223	90.47 ± 11.67 **
Cholesterol (mg/dL)	359	167.48 ± 32.79	218	168.61 ± 30.70
HDL (mg/dL)	359	52.02 ± 11.60	218	40.95 ± 11.14 **
LDL (mg/dL)	359	96.02 ± 28.61	218	103.75 ± 27.71 ***
Fasting insulin (uIU/mL)	378	5.37 ± 5.26	223	6.09 ± 5.76
Mean BP ^	372	85.52 ± 8.75	218	90.17 ± 9.21 **
HOMA ^^	378	1.16 ± 1.49	223	1.40 ± 1.48
Subcutaneous fat volume of the trained arm (mm^3^)	273	260697 ± 119542	146	181908 ± 97743 **
Subcutaneous fat volume of the untrained arm (mm^3^)	273	261558 ± 122587	146	184542 ± 105259 **

*p = 0.003

**p < 0.0001

*** p = 0.0015

^Mean BP calculated as (SBP + DBP*2)/3

^^ HOMA calculated as ((Fasting glucose (mg/dL) * 0.0551 * Fasting insulin (uIU/mL))/22.5)

### Exercise Training Program

Resistant training was performed with the non-dominant arm. The protocol was described elsewhere [24]. Briefly it consisted of two 45 – 60 minute sessions per week for 12 weeks. Each session was supervised by an exercise physiologist professional or a trained student. Before each session, participants warmed-up with 2 sets of 12 repetitions of the biceps preacher curl and the seated overhead triceps extension. Each session included dumbbell biceps curls, dumbbell biceps preacher curls, and incline dumbbell biceps curls, overhead dumbbell triceps extension, and dumbbell triceps kickbacks. The amount of weight was aggressively increased during the 12 wks.

### Subject Phenotyping

#### Anthropometric assessment

Body weight was recorded before and after the exercise training using a balance beam scale. Height was measured using a tape mounted on a wall and recorded in inches. BMI was calculated from weight (kg) and height (m).

#### MRI assessment

Subjects were scanned in the supine position with arms at their sides and their palms up on the scanner bed surface. The arm maximum circumference was determined with the subject standing, with the shoulder abducted at 90 degrees, the hand supinated, and the biceps flexed. A vitamin E bead was placed on the front of the biceps portion of the arm with the largest circumference to standardized MRI measurements by comparing the bead's measured cross sectional area with that of the MRI determined cross sectional area.

Entry MRI was done 24 to 48 h before the isometric or 1RM (1 repetition maximum) test. Scans post-training MRI was performed 48 – 96 h after the last training session. Fifteen 16 mm contiguous axial slices from each arm were taken from each arm independently. The top of the bead in a sagittal scout view was used to locate the 8^th ^slice going from the top of the arm toward the elbow. Scans for both arms were taken by Fast Spoiled Gradient Recalled (FSPPGR) and Fast Spin Echo (FSE) with TE 1.8/TR 200 msec. All 8 centers submitted the MRI data to the Research Center for Genetic Medicine at Children's National Medical Center (CNMC) in Washington, D.C, via e-mail or DAT disc, and all scans were integrated into the study SQL database.

**Figure 1:**
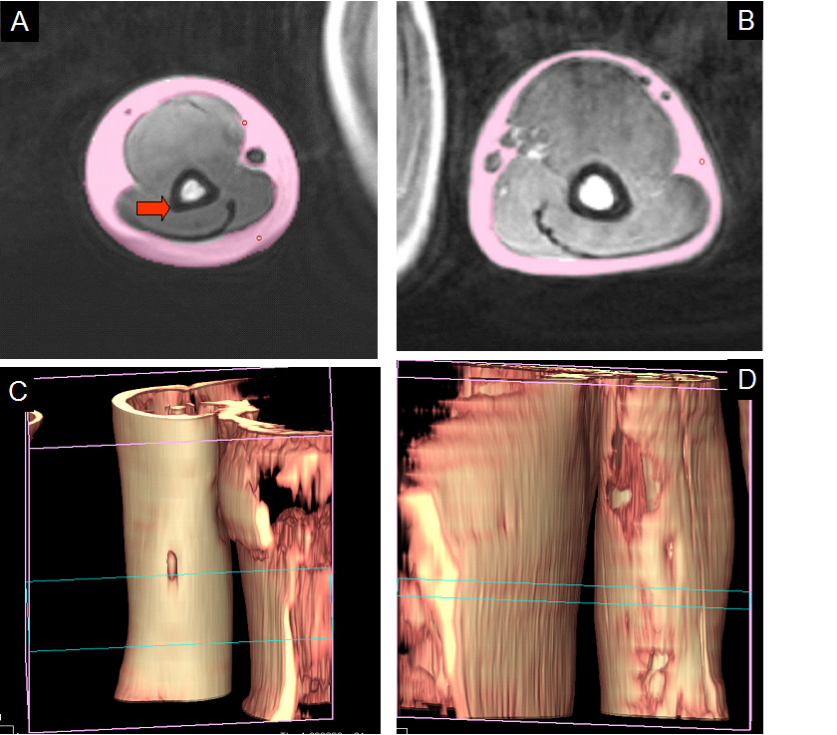
**3D determination of fat volume**. Rapidia determination of subcutaneous fat volume. Panel A: the pink selected area shows the subcutaneous fat of one of the six slices measured in the right arm. The red arrow shows the epypheseal flare (anatomical landmark used as the starting point of fat determination). Panel B: Left arm, the pink area shows the subcutaneous fat volume for other subject. Panel C and D represent the 3D image of panel A and B.

**Figure 2:**
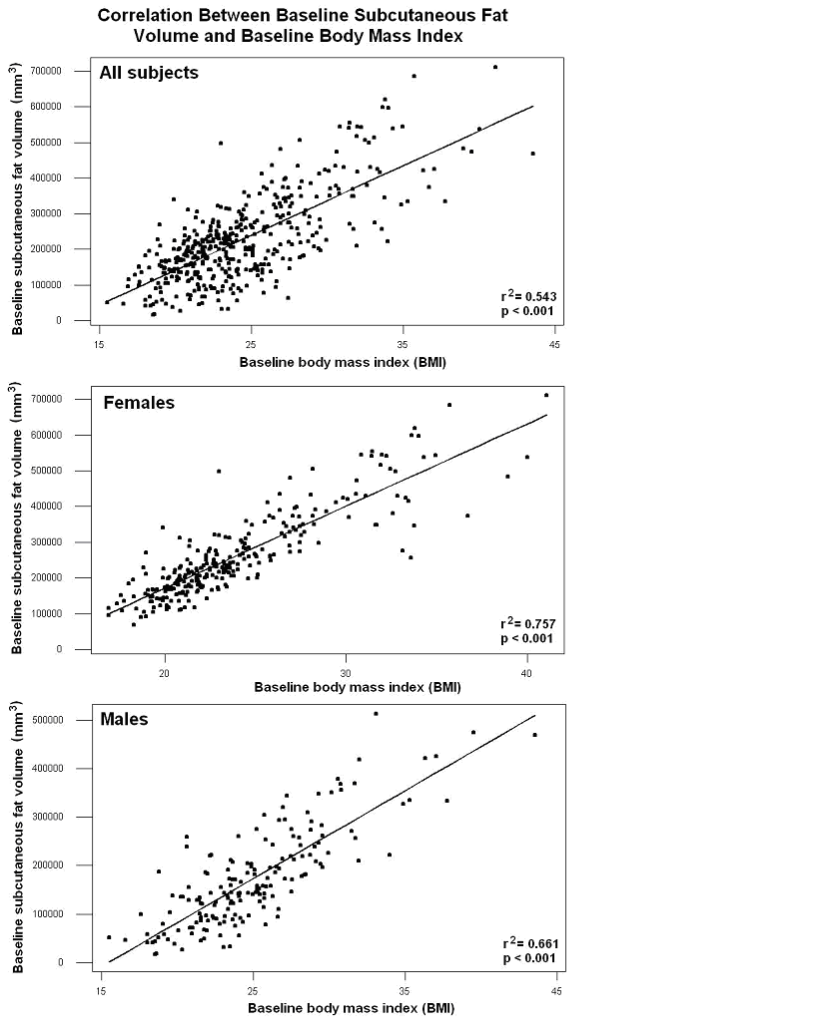
**Pearson correlation between baseline and post-training subcutaneous fat in the untrained arm of the entire cohort**. The correlation coefficient between baseline and post exercise subcutaneous fat volume in the untrained-arm was R^2 ^= 0.943 (P = 0.001).

For both cross-sectional and volumetric analysis of the MRI images, we used Rapidia (INFINITT Inc, Seoul Korea), a PC based software that allows the semi-automatic quantification of muscle, bone and subcutaneous fat (figure 1). The software was optimized to provide automated position adjustment, and to distinguish muscle, fat, and bone, with automatic edge detection, user modification of ambiguous edges, and automated propagation of defined tissue boundaries when possible. Volume measures were taken using an anatomical landmark (metaphyseal-diaphyseal junction of the humerus) as our starting point and assayed the six 1 cM slices proximal to it. Reliability of measures was determined using the untrained arm, with fat volume measured at a 12 wk interval, showing that our quantitation of subcutaneous fat was highly reliable and sensitive (R^2 ^= 0.943) (figure 2). Measurement values were automatically written and saved in a SQL database together with anthropomorphic and genotyping data.

### Serum biochemistry

Venous blood samples were collected from fasting subjects and serum was separated from blood cells by centrifugation at 1100 × g for 10 min and frozen for further analysis. Serum samples were analyzed by QuestDiagnostic.

### DNA extraction and genotyping

DNA was extracted from blood samples obtained by phlebotomy before starting the exercise training. Genotyping was done using the TaqMan allele discrimination assay that employs the 5' nuclease activity of Taq polymerase to detect a fluorescent reporter signal generated during PCR reactions. Oligonucleotide primers used for the L163V PPARα assay are LVF: CAGAAACAAATGCCAGTATTGTCGAT and LVR: CTTACCTACCGTTGTGTGAC ATC. LVV2 VIC: ACAAGTGCCTTTCTG (G allele) and LVM2 AM: CAAGTGCGTTTCTG (C allele)

### Statistical analyses

The Hardy-Weinberg equilibrium was determined for each SNP using a χ^2 ^test to compare the observed genotype frequencies to those expected under H-W equilibrium.

Three volumetric measurements were analyzed as continuous quantitative traits (baseline and post-exercise subcutaneous fat volumes and difference in subcutaneous fat volume from baseline to post exercise) for each arm. Normality of each quantitative trait was confirmed using the Shapiro-Wilk normality test.

Bivariate correlation analyses of each quantitative measurement showed several significant correlations with age and baseline mass, therefore, associations between each SNP and volumetric measurements were assessed using analysis of covariance (ANCOVA) methods. Due to large gender differences in baseline values and the response to training, all analyses were performed separately for men and women. All significant associations from the main ANCOVA model were subjected to pair-wise statistical tests among each of the three genotype groups for each SNP. Linear tests were performed between each of the genotype groups to determine which genotype groups were significantly different from one another. The resulting p-values from these linear tests were adjusted for multiple comparisons using the Sidak post-hoc multiple comparison test. Linear regression analysis, including likelihood ratio tests between full (containing genotype and covariates) and constrained (containing covariates only) models, were performed to estimate the proportion of variance in volumetric measurements attributable to each SNP's genotype.

## Results

### Genotype association of PPARα L162V polymorphism with lipid concentrations

610 Caucasian subjects were selected for this study, based upon availability of serum samples for lipid and glucose determination (n = 578), and/or complete MRI data (n = 526) (Table 1). All subjects were genotyped for the PPARα L162V polymorphism using automated TaqMan assays. Genotype distribution was in Hardy-Weinberg equilibrium. The allele frequencies of the PPARα L162V showed the rare Valine allele at 7.5% in Caucasians, with18% of Caucasians heterozygous for the allele. This is similar to previous reports in other Caucasian populations.

The PPARα 162 valine allele was associated with significantly greater serum triglyceride and lower HDL-chol levels in Caucasian men. Heterozygotes for L162V showed 78% higher triglyceride levels than homozygotes for the common ancestral allele (n = 578; LL genotype = 116.15 ± 10.82 mg/dL LV genotype = 207.49 ± 29.49 mg/dL; p = 0.004) (Table 2). As 18% of the population carried the rare allele, the genotype effect of the SNP for all variation in triglyceride levels was approximately 3.8%. This was a larger effect size than previously reported for any genetic modifier of serum triglyceride levels.

**Table 2:** Analysis of Serum measurements and PPAR alpha (L162V) in Caucasians

**Serum Measurement**	**Group**	**Covariates**	**P-value**	**N; Adjusted mean ± SEM**	**p-value for significantly different means**	**% variation explained by genotype; LRT p-value**
Triglycerides	All subjects	Age	0.0026	CC (N = 500; 102.92 ± 4.49)* CG (N = 71; 146.80 ± 11.84)* GG (N = 7; 114.99 ± 37.98)	*p = 0.0018	2.0%; 0.0025
	Females	Age	0.0474	CC (N = 307; 94.76 ± 2.59)* CG (N = 45; 111.11 ± 6.77)* GG (N = 7; 114.72 ± 17.17)	NONE	1.7%; 0.0458
	Males	Age	0.0040	CC (N = 193; 116.15 ± 10.82)* CG (N = 26; 207.49 ± 29.49)*	*p = 0.0040	3.8%; 0.0037
HDL	All subjects	Age	0.0076	CC (N = 500; 48.13 ± 0.56) CG (N = 70; 44.67 ± 1.48)* GG (N = 7; 58.43 ± 4.74)*	*p = 0.0173	1.7%; 0.0073
	Females	Age	NS			
	Males	Age	0.0017	CC (N = 193; 41.77 ± 0.78)* CG (N = 26; 34.54 ± 2.13)*	*p = 0.0017	4.4%; 0.0015
Cholesterol	All subjects	Age	NS			
	Females	Age	NS			
	Males	Age	NS			
VLDL-TG	All subjects	Age	NS			
	Females	Age	0.0441	CC (N = 307; 18.94 ± 0.52)* CG (N = 45; 22.29 ± 1.35)* GG (N = 7; 22.77 ± 3.43)	NONE	1.7%; 0.0426
	Males	Age	NS			
LDL	All subjects	Age	NS			
	Females	Age	NS			
	Males	Age	NS			
Fasting glucose	All subjects	Age	NS			
	Females	Age	0.0440	CC (N = 307; 85.78 ± 0.41) CG (N = 45; 83.65 ± 1.03) GG (N = 7; 89.71 ± 2.58)	NONE	1.5%;0.0426
	Males	Age	NS			
Fasting insulin	All subjects	Age	NS			
	Females	Age	NS			
	Males	Age	NS			

LDL cholesterol levels tended to be higher among carriers of the V162 allele, although these results did not reach statistical significance (LL genotype = 96 mg/dL LV genotype = 98 mg/dL).

Heterozygous men showed 20% lower HDL levels than homozygotes (LL genotype = 41.77 ± 0.78 mg/dL LV genotype = 34.54 ± 2.13 mg/dL p = 0.001).

No significant association between PPARα genotype and lipids in women, for any subtype of lipid (Table 2).

### Genotype association of PPARα L162V polymorphism with insulin resistance

We calculated insulin resistant using homeostatic model assessment (HOMA-IR), where the product of fasting glucose (mmol/L) and fasting insulin (μU/mL) was divided by 22.5. No significant differences were seen for insulin resistance and the 162V polymorphism in our cohort.

### Genotype association of PPARα L162V polymorphism with baseline subcutaneous fat volume

Fat volume was determined using semi-automated three-dimensional reconstruction analysis software (Figure 1), with anatomical location specified by the epiphyseal flare of the humerus. Fat volume was measured for both upper arms, before and after a 12 week supervised resistance training program implemented only on one arm. Reliability of measures was determined using the untrained arm, with fat volume measured at a 12 wk interval, showing that our quantitation of subcutaneous fat was highly reliable and sensitive (R^2 ^= 0.943) (figure 2). We considered six phenotypes for association with PPARα L162V: absolute fat volume baseline (dominant and non-dominant arms), absolute fat volume after unilateral strength training intervention (both dominant, and non-dominant [trained] arms), and absolute change in fat volume (both dominant and non-dominant arms). All data were stratified for sex (Table 3).

**Table 3:** Analysis of PPAR alpha (L162V) in larger Caucasian only MRI cohort:

**Subcutaneous fat measure**	**Gender**	**SNP**	**N: adjusted mean ± SEM**	**P-value for significantly different means**	**Variability attributable to genotype**	Likelihood-ratio test p-value *
**Full model – with age only adjustment**						

Trained arm – baseline fat volume	Male	PPAR alpha (L162V)	CC (N = 179; 167221 ± 6710)* CG (N = 24; 207418 ± 18325) *	* p = 0.041	2.0%	0.039
Trained arm – post-exercise fat volume	Male	PPAR alpha (L162V)	CC (N = 179; 170608 ± 6952)* CG (N = 24; 214926 ± 18932) *	*p = 0.029	2.3%	0.028
Untrained arm – baseline fat volume	Male	PPAR alpha (L162V)	CC (N = 179; 169890 ± 7106)* CG (N = 24; 224144 ± 19407) *	*p = 0.033	2.2%	0.031
Untrained arm – post-exercise fat volume	Male	PPAR alpha (L162V)	CC (N = 179; 168183 ± 7241)* CG (N = 24; 231960 ± 19776) *	*p = 0.003	4.2%	0.003
Untrained arm – difference in fat volume	Male	PPAR alpha (L162V)	CC (N = 179; -1707 ± 2133)* CG (N = 24; 17617 ± 5826) *	*p = 0.002	4.6%	0.002

**Full model – with age and weight adjustment**						

Untrained arm – difference in fat volume	Male	PPAR alpha (L162V)	CC (N = 179; -1921 ± 2134)* CG (N = 24; 17612 ± 5825) *	*p = 0.002	4.9%	0.001

The PPARα 162 valine allele was associated with significantly greater baseline subcutaneous fat volume in Caucasian (Table 3). Four different phenotypes all showed significant genotype effect for absolute measures of fat volume (trained, un-trained, entry and exit; Table 3). Male heterozygotes for L162V showed 24% to 38% higher subcutaneous fat of the arm than homozygotes for the common ancestral allele (LL genotype = 162,106 mm^3^; LV genotype = 256,388 mm^3^). As 18% of the population carried the rare allele, the genotype effect of the SNP for all variation in regional baseline subcutaneous fat volume is men was approximately 2% to 4.2% ; Table 3).

We also examined the association between PPARα and BMI in Caucasian men (as an independent variable). The Valine allele was associated with higher BMI in Caucasian men (P = 0.0031) (LL genotype = 25.10, LV genotype = 27.54) (n = 202).

For women, our analysis did not show any statistically significant genotype effect for subcutaneous fat.

### Genotype effect on exercise-induced changes in subcutaneous fat volume

**Figure 3:**
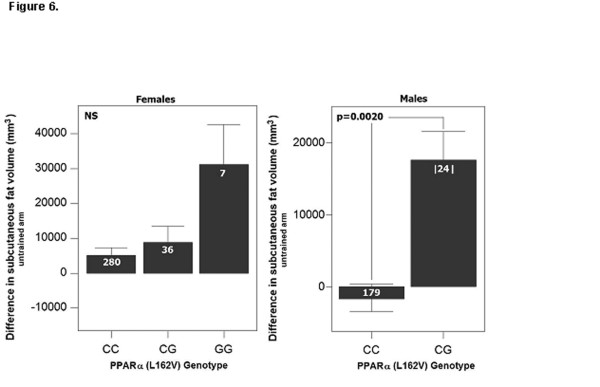
**PPARα L162V is associated with changes in subcutaneous fat volume in response to exercise in men**. In men heterozygote for the V162 exercise training increased fat volume of the untrained arm. LL genotypes significantly decreased in fat volume. No significant differences were seen for women. Aged adjusted model.

The PPARα 162 valine was associated with increased fat volume of the untrained arm in male carriers, while LL genotypes significantly decreased in fat volume (LL = -1,707 ± 21 mm^3^, LV = 17,617 ± 58 mm^3^; p = 0.002) in men (figure 3; table 3). This suggests that exercise results systemic loss of subcutaneous fat in LL males, while the LV carriers showed a large gain in subcutaneous fat.

As with baseline genotype associations, we found no association in women for change of subcutaneous fat as a function of exercise.

## Discussion

The major goal of this study was to identify the role that PPARα L162V polymorphism may play in the variability of serum lipid levels, BMI, subcutaneous fat volume, and change in fat volume in response to unilateral upper arm resistance training in 610 young (24 yrs) healthy volunteers.

We found the V allele to increase triglycerides by 78% (p = 0.004), and this single polymorphism accounted for 3.8% of all variation in serum triglycerides in males (p = 0.0037). PPARα L162V has been previously shown to be associated with cholesterol [19] LDL-chol [16-18], HDL-chol [18] or triglycerides levels [20,21] depending on the population studied. However, the majority of these association studies have not been replicated. For example, the effect of the PPARα L162V polymorphism on lipid profiles has been studied in middle age subjects with and without endo-phenotypes of metabolic syndrome in three different reports. Vohl et al. [17] reported 10% higher LDL cholesterol levels in carriers of the V allele in non-diabetic men (n = 193), but not in a diabetic population of men and women (n = 120) (p = 0.02). In contrast, Flavell et al. have found higher levels of LDL cholesterol in carriers of the V allele in subjects with type 2 diabetes (n = 129) but not in healthy subjects (n = 2508) [18]. Moreover, Lacquemant et al. reported higher cholesterol levels in subjects with metabolic syndrome only in the presence of concomitant coronary heart disease (CHD) [19]. The discrepancies among these studies may be the result of effect modifiers in different populations, with disease state, advanced age, and sex each potentially modulating genotype/phenotype associations. Our underlying rationale in this current study was that analysis of young pre-morbid normal populations would provide the most sensitive and specific window into PPARα associations with lipid profiles. Consistent with our hypothesis, the association of PPARα and triglycerides in males in this current study showed a much larger effect of the V allele than previously reported in older and less healthy populations, with the rare V allele nearly doubling circulating triglycerides.

Our data showed that the V allele resulted in a 24–38% increase in subcutaneous fat, and a 20% increase in BMI (p = 0.0031) (CC genotype = 25.10 kg/m^2^, CG genotype = 29.54 kg/m^2^) in White males. We also found that the PPARα polymorphism had a strong effect on change in subcutaneous fat volume after 12 wks of unilateral resistance training in males. Specifically, males homozygous for the common L allele showed a decrease in fat volume following unilateral resistance training, while V allele heterozygotes showed an increase in subcutaneous fat (p = 0.002). The effect was on both the trained and untrained arms, suggesting that the PPARα genotype had a strong systemic effect on subcutaneous fat metabolism, where the L allele homozygotes showed a beneficial effect of exercise on adiposity, while the V allele carriers put on weight with exercise. To our knowledge, this is the first demonstration of the effect of PPARα genotype on the outcome of an intervention.

While PPARα Valine allele showed strong statistical significance with higher subcutaneous fat and BMI in young Caucasian men in our study, the two previous reports showed association of the Valine allele with lower percentage of body fat, and lower BMI, in overweight and diabetic women [22,23]. A possible explanation for this apparent discrepancy is as follows. Functional studies on PPARα L162V have reported a higher transactivation of the receptor in the presence of the variant allele (162V) in a ligand dependant way [15]. At low ligand levels, the activity of 162V receptor was lower than the wild type. However, at high ligand levels, the activity of the polymorphic receptor was higher. Since the activity of the receptor is in part dictated by the concentration of the receptor agonists and PPARα is activated by fatty acids among others, we would expect to see higher activity of the polymorphic receptor in obesity, metabolic syndrome and type 2 diabetes (in which the levels of fatty acids are higher). Levels of endogenous and exogenous ligands (such as dietary fatty acids) may dictate the final phenotype observed in the presence of the polymorphism. Our data in young pre-morbid subjects on lipid levels, BMI and subcutaneous fat volume is consistent with the 162V allele showing lower PPARα activity.

One finding of our study is that women do not show the same PPARα L162V associations as do men. It is clear that gender greatly influences both PPARα expression, and also use of alternative lipid metabolism pathways, and that both are highly hormone responsive. Djouadi et al. [25] reported that in PPARα null mice the pharmacological inhibition of fatty acid cellular flux caused massive hepatic and cardiac lipid accumulation, hypoglycemia (secondary to reduced gluconeogenesis due to the inhibition of lipid β oxidation), and death in 100% males but only 25% females. The pretreatment of males with estrogens abolished the difference between the two sexes. In other studies, Jalouli et al. [26] found higher PPARα mRNA and protein levels in male rats than in female rats. Gonadectomy of male rats reduced the expression of PPARα to similar levels as intact female rats. This suggested that not only estrogens may have a role in the regulation of lipid metabolism but also androgens may be involved through the increased expression of PPARα. Taking these results together, young females with circulating estrogen levels appear to have alternative lipid metabolism pathways that would serve to mask the effect of the PPARα polymorphism, as we have found in our young adult populations.

It is important to note that the relatively rare frequency of the V allele leads to small group sizes of V carriers. Future studies using specific interventions on pre-genotyped groups of V allele carriers will be necessary to more fully characterize the effect of the PPAR V allele on metabolism and measures of obesity.

## Conclusion

We found statistical support for a well characterized PPARα polymorphism (L162V) with plasma triglycerides, HDL, subcutaneous fat volume, and change in fat following an exercise intervention in 610 Caucasian young healthy volunteers. Those Caucasian men carrying the 162V allele have nearly double serum triglyceride levels, lower HDL levels, greater regional subcutaneous fat volume, higher BMI, and tend to gain subcutaneous fat volume with an exercise intervention.

## Competing interests

The author(s) declare that they have no competing interests.

## Authors' contributions

JU performed the genotyping and draft the manuscript. HGD carried out statistical analysis. MB participated in genetic conselling. BM helped to genotype. CTR, JD, BH, EKR and CB carried out the quantification of fat volume. BCH helped to draft the manuscript. RLS, PDT, TBP, TJA, PMC, NMM, LSP, PSV, RFZ and PMG participated in the design of the study. EPH conceptualized, designed the study and drafted the manuscript. All authors read and approved the final manuscript.

## Pre-publication history

The pre-publication history for this paper can be accessed here:


